# ‘*Emigration is a matter of self-preservation. The working conditions . . . are killing us slowly*’: qualitative insights into health professional emigration from Ireland

**DOI:** 10.1186/s12960-015-0022-6

**Published:** 2015-05-16

**Authors:** Niamh Humphries, Sara McAleese, Anne Matthews, Ruairi Brugha

**Affiliations:** Department of Epidemiology and Public Health Medicine, Royal College of Surgeons in Ireland, RCSI, 123 St. Stephen’s Green, Dublin 2, Dublin, Ireland; School of Nursing and Human Sciences, Dublin City University, Dublin, Ireland

## Abstract

**Background:**

Achieving a sustainable health workforce involves training and retaining sufficient staff to deliver health services. The Irish health workforce is characterised by a high level of emigration of Irish-trained staff and a heavy reliance on internationally trained staff. This paper presents qualitative findings from a mixed-method study of doctors, nurses and midwives who have recently emigrated from Ireland.

**Methods:**

Using Facebook, this study elicited 556 (388 completed) responses to an exploratory mixed-method online survey in July 2014. Respondents provided rich responses to two free-text questions, one on health worker return (*N* = 343) and another on health professional emigration (*N* = 209) from the source country (Ireland).

**Results:**

Respondents emigrated because of difficult working conditions in the Irish health system (long working hours, uncertain career progression), which compared poorly with conditions in the destination country. Respondents’ experiences in the destination country vindicated the decision to emigrate and complicated the decision to return. Their return to Ireland was contingent upon significant reform of the Irish health system and an improvement in working conditions, expressed, for example, as:

‘*It’s not about the money, it’s about respect . . . we love working in medicine, but we love our families and health more*’ (RD283).

**Conclusions:**

This paper highlights that doctors, nurses and midwives are emigrating from Ireland in search of better working conditions, clear career progression pathways and a better practice environment. The question for the source country is whether it can retain and attract back emigrant doctors, nurses and midwives by matching their expectations.

## Background

### Health professional emigration

A key component of a sustainable health workforce is the ability to ‘*keep scarce skills in the system by effective retention strategies*’ [1]. Despite the importance of retention for source countries, most collect little or no data on the emigration of health professionals. Alongside a dearth of data on health professional emigration, little is known about emigrant health professionals, their motivations for emigration and level of interest in return. This information is important in facilitating better understanding of, and responses to, health professional emigration by the source country. As Kapur explains, ‘*citizens leave their country for a reason. And when they leave, the factors that led them to leave do not disappear. Understanding these factors is critical*’ [2], both to their retention and also to attracting them back to the source country.

Research on health professional migration in high-income countries has tended to focus on them as destination rather than source countries. The few studies that have considered the factors influencing the emigration of health professionals from European Union (EU) source countries offer valuable insights. Research has found that United Kingdom (UK)-trained doctors in New Zealand emigrated primarily for professional reasons, such as a desire for improved training and career progression [3]. German doctors’ intent on emigration cited heavy workloads, poor working conditions and poorly structured postgraduate training as factors influencing the emigration decision [4]. UK-trained midwives who migrated to Australia noted that they had felt overworked and stressed and had struggled to provide good care in the UK [5]. German emigrant nurses cited dissatisfaction with working conditions, low remuneration and professional recognition as reasons for their emigration [4].

### Health professional emigration in the Irish context

Since 2008, Ireland has become one of the EU countries hardest hit by economic recession and has experienced higher levels of general emigration than countries such as Spain or Greece [6]. All indications suggest high levels of health professional emigration from Ireland since the onset of economic recession in 2008 [7, 8]. Some initial indications of the impact of health professional emigration on the Irish health system include:

- A 100% increase in spending on agency or locum junior doctors between 2013 and 2014 [9].
- Although the total vacancy rate for non-consultant hospital doctor (NCHD) posts as of January 2015 was small (177 of 5300) (personal communication, Health Service Executive (HSE)), most of these are likely to be posts in rural hospitals and those not connected to formal postgraduate training schemes.
- A decrease of 12 000 in the number of directly employed whole-time equivalent staff since a peak in 2007 and significantly increased spending on agency staff in recent years [10, 11].
- Health employers struggling to fill nursing posts in surgical theatre and critical care specialties [12].
- Warnings from the Irish Medical Organisation^a^ and from general practitioners of an emerging staffing crisis in the Irish health system as a result of doctor emigration [13, 14].

However, in Ireland, as in other developed countries, there are limited data available to confirm outward health professional migration trends [15–17]. The main source of data on the medical workforce comes from the medical register, which records the number of doctors *registered* to practise, rather than the number active in the medical workforce (Table 1). There has been an increase in the number of hospital doctors employed in the public sector since 2008 as a result of the continued implementation of the European Working Time Directive (EWTD). No disaggregation of these data is available. While 34.3% of doctors registered in Ireland are internationally trained [18], the number of internationally trained doctors entering the workforce annually or employed in the public sector is unknown. As a result, the inward migration of internationally trained doctors may mask the scale of doctor emigration from Ireland, as posts vacated by emigrating Irish trained doctors are filled by internationally trained doctors.

**Table 1:** **Data on doctors registered/employed in Ireland, 2008–2014** ^**b,c**^ [14 **,** 28 **,** 50] **(personal communication, HSE, Medical Council)**

	**2008**	**2009**	**2010**	**2011**	**2012**	**2013**	**2014**	**2015**
Number of doctors on register	17 741	18854	18 770	18 812	18 184	18 160	19 066	19 616
Number of public sector consultants and junior hospital doctors (NCHDs) employed (headcount)	7 197	7 120	7 127	7 413	7 418	7 474	7 862	8 554
Number of public sector employed, medical/dental	8 109	8 083	8 096	8 331	8 320	8 353	8 713	9 761
Number of general practitioners (GPs) contracted to HSE (2010 and 2014)			2 258				2 416	

Table 2, which presents data on the numbers of nurses and midwives registered in Ireland, also shows a significant discrepancy between the number registered to practise and those employed in the nursing/midwifery workforce (public sector). Although there was an increase in the total number of nurses registered to practise between 2008 and 2014, the number employed in the public sector has decreased by 11% during that time (see Table 2). This decrease could be largely considered a result of austerity-related measures such as the incentivised early retirement schemes for public sector staff, as well as a result of emigration and perhaps as a result of a move of staff into the private healthcare sector, which is not reliably measured nationally. The nursing register allows registered nurses and midwives to classify themselves as active or inactive in the Irish health workforce. As Table 2 shows, the number registered on the active register decreased by 6% between 2008 and 2014.

**Table 2:** **Data on nurses registered/employed in Ireland, 2008–2014** [50, 51]

	**2008**	**2009**	**2010**	**2011**	**2012**	**2013**	**2014**
Number of nurses/midwives on register	88 224	89 504	90 530	91 700	92 726	94 715	94 604
Number of nurses/midwives on active register	68 614	68 483	67 415	67 130	66 888	66 409	64 790
Number of nurses employed in the public sector	38 108	37 466	36 503	35 902	34 637	33768	33 992

#### Verification data

To emigrate as a health professional and practise in another country, the registration body in the destination country must verify the good standing of the health professional with the registration body in the source country, a process known as verification. Professional registration bodies collect data on the numbers of verification requests annually, and this is the primary indicator of health professional emigration intent in the Irish context [15, 19]. Although valuable as a source of data on intent to emigrate, the difficulties with verification data are multiple: health professionals may emigrate without applying for verification, they may apply for verification retrospectively (after emigration has already occurred), they may apply for verification on several occasions, or they may apply and not follow through with emigration [17]. Verification data may be subject to manipulation as health professionals can use verification requests to pressurise employers and/or governments to initiate debate on health professional emigration or working conditions [17]. Verification data are perhaps best interpreted as indicative of a health professional’s *intent* to emigrate rather than a measure of *actual* emigration [15, 16]. Despite the limitations of verification data, it is currently one of the only methods of measuring health professional emigration from Ireland.

**Figure 1:**
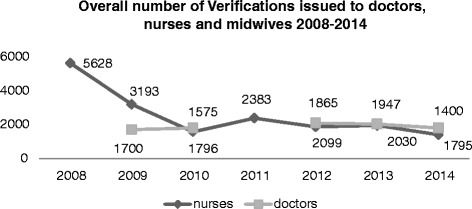
Overall number of verifications issued to doctors, nurses and midwives, 2008–2014.^d^ Source: data from MCI and NMBI.

**Figure 2:**
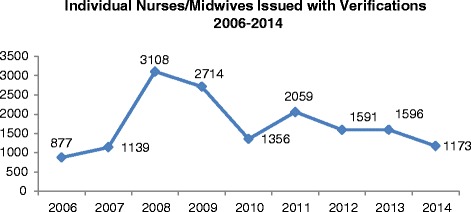
Individual nurses/midwives issued with verifications, 2006–2014. Source: data from NMBI.

Figures 1 and 2 present verification data obtained from the Medical Council of Ireland (MCI) and the Nursing and Midwifery Board of Ireland (NMBI). The data presented in Figure 1 relate to the overall number of verification certificates issued, rather than the number of individual health professionals issued with verification certificates (these data are unavailable for doctors in the Irish context). In 2013 when 500 doctors acquired verification from the GMC, it was described as an exodus and a significant danger to the UK medical workforce [20]. Figure 1 indicates that Ireland may be experiencing quite a significant level of doctor emigration relative to the size of its medical workforce.

Figure 2 shows the number of individual nurses/midwives issued with verification certificates between 2006 and 2013. Interesting in this context is the impact that an external shock, such as the onset of economic recession in 2008/9, appears to have had on nurses/midwives’ intent to emigrate, with record number of verifications issued in those years (Figure 1), equating to approximately 3000 individual nurses/midwives in each of those years (Figure 2). In 2009, Ireland graduated approximately 1264 nurses/midwives [16]. The number of verifications issued has since levelled off somewhat and approximately 1200–1500 nurses and midwives per annum obtain verification certificates (Figure 2). This includes non-EU migrant nurses and midwives [16] as well as Irish-trained nurses/midwives and suggests a potentially significant outflow from the Irish health system, albeit a flow that appears to be stabilising.

#### Destination country data

Another means of measuring the rate of health professional emigration from Ireland is by examining the immigration or registration records of destination countries. Improved information sharing internationally on health professional migration is a recommendation of the World Health Organization (WHO) Global Code of Practice on the International Recruitment of Health Personnel [21] and represents an opportunity for source country emigration trends to be verified with destination countries. By way of example, Table 3 presents data on the inward migration of Irish-trained doctors and nurses to Australia and indicates a significant flow of nurses and doctors from Ireland to Australia between 2009 and 2014. Although most entered Australia on temporary entry visas, there is scope for health professionals to switch to more permanent visas once in-country.

**Table 3:** **Irish-trained health professionals issued with Australian visas, 2009–2014** [52]

	**Permanent entry visas**	**Temporary entry visas**	**Total**
Irish-trained doctors	155 doctors	1000 doctors	1155 doctors
Irish-trained nurses	328 nurses	2010 nurses	2338 nurses

### Nurses/midwives in Ireland

According to Organisation for Economic Co-operation and Development (OECD) figures, Ireland has 12.6 nurses per 1000 population in comparison to the EU28 average of 8 nurses per 1000 population [22]. However, as outlined above, these figures relate to the number of nurses/midwives on the active register^e^ rather than the number in direct nursing/midwifery practice [23]. There are no national nursing/midwifery workforce databases that measure the overall numbers of nurses/midwives in practice and therefore no national data on nurse–patient ratios or on working patterns. The RN4CAST study of nurse staffing in 12 European countries estimated a patient-to-nurse ratio in Irish medical/surgical wards of 6.9:1 [24], which was the second lowest (i.e. most favourable) in the study [24]. However, the study also found that nurses in these wards in Ireland worked an average shift of 12 h, which is not the pattern in RN4CAST, where 50% of nurses worked shifts of 8 h or less [25]. The RN4CAST data only apply to general medical/surgical wards in acute adult hospitals and so are not representative of the entire nursing workforce. Ireland’s nursing/midwifery workforce has been impacted by austerity-related measures to reduce the public sector wage bill, namely the public sector recruitment embargo and incentivised early retirement schemes [16].

In Ireland, as internationally, austerity-related savings were sought via a reduction in staff numbers rather than by improving efficiency in the health system [26]. Although the number of nurses/midwives employed in the public sector has decreased since 2009 (see Table 2), there has been a marked increase in hospital activity (inpatient and day), meaning that fewer staff treat a greater number of patients [10]. The public sector recruitment embargo 2009–2013 meant there were few employment opportunities open to newly qualified nurses and midwives. A graduate nurse employment scheme launched in 2013 offering 1000 new nursing positions at reduced salary rates was controversial and was the focus of a boycott by the Irish Nursing and Midwifery Organisation^f^ (INMO). Given the bleak employment prospects, particularly for newly qualified nurses and midwives since 2009, high emigration levels are unsurprising and the available data illustrate this.^g^ A national survey of the nursing and midwifery graduates in 2010 found that 29% (179/610) were working abroad within a year of graduation [27]. Irish-trained nurses and midwives have been actively recruited by employers in the UK, United States of America (USA), Canada and Australia in recent years.

### Doctors in Ireland

Ireland has 2.7 doctors per 1000 population, according to OECD figures [22], compared to the EU28 average of 3.4 doctors per 1000. However, this ratio is based on the number of doctors registered, which may be greater than the number in the workforce^h^ [28]. Table 1 shows some discrepancy between the number of doctors registered and the numbers in public practice. Some of the discrepancy can be explained by the employment of doctors in the private healthcare sector, for which reliable workforce data are not available.

Accounts in the media by Irish-trained doctors who have emigrated refer to poor working conditions and the pressure of working in the Irish health system, describing it as a system ‘*that was quite obviously failing*’ [29] as a reason for their emigration. In terms of working hours, Ireland has struggled to achieve EWTD compliance. Hospital doctors frequently work more than the mandated 48 h per week, with recent research reporting that junior hospital doctors typically working 80–90 h per week [30]. A recent campaign by junior hospital doctors sought to have hospital shifts restricted to 24 h in duration.

Emigrant Irish-trained doctors also cite ‘*inadequate training, inadequate remuneration and lack of career prospects*’ [31] as factors influencing their decision to emigrate. This relates to the postgraduate training structures in Ireland. Having completed their medical degree, doctors in Ireland embark on postgraduate specialist training of between 4 and 12 years in duration (depending on specialisation). Throughout their training, doctors are employed on temporary contracts and generally move hospital/location every 6–12 months [32, 33], although proposals to provide greater predictability of location for doctors on training schemes are being introduced [34]. Once this training has been successfully completed, doctors are eligible to apply for (but are not guaranteed) a specialist post of Consultant or General Practitioner within the Irish health system.

Although no comprehensive data on doctor emigration from Ireland are currently available, the available data indicate substantial levels of emigration. For instance, a survey which tracked doctors who completed internship^i^ in the Irish health system in mid-2011 found that 45% were no longer working in the public health system in Ireland, and it is likely that the majority had emigrated [7]. Exits from the Irish medical register are also an indication of emigration. In 2012, exit rates of 6.4% and 6.3% were recorded for doctors aged 25 to 29 years and 30 to 34 years [18], and similar rates were recorded in 2013 with 7.9% of doctors aged 25-29 years and 6% of doctors aged 30–34 exiting the medical register [28]. A general limitation of professional register exit data is that it does not differentiate between exit for emigration and exit for other reasons (retirement, death, change of profession, etc.); however, such high exit rates in younger cohorts suggest emigration as a major factor. A further limitation is that health professionals may maintain registration in their home/source countries while working abroad.

### Health professional emigration and health workforce planning

To develop a sustainable health workforce, as recommended by the WHO Global Code of Practice on the International Recruitment of Health Personnel [21], each country must ‘*educate, retain and sustain*’ an appropriate health workforce [21]. Ireland has recently scaled up its medical training and is educating sufficient numbers of health professionals to meet demand [35]. Yet a failure to sustain and retain those health professionals has resulted in emigration and a continued reliance on internationally trained health professionals to staff the health system.

The evidence base and systems for routine monitoring of health professional emigration, needed for the development of ‘*effective health workforce policies and planning*’ [36], are weak in the Irish context. While national media highlight the problem, minimal systematic evidence has been generated around health professional emigration. Could Ireland’s emigrant health professionals be considered ‘*crisis escapees*’ [37] whose emigration is a direct result of the recession, or as a result of recession-related cost-containment in the health sector [26]? This scenario might see emigration patterns mirror Ireland’s economic situation. Alternatively, emigration may be a result of sudden ‘triggers’ [38] or as a result of a more gradual build-up of frustration by health professionals [38], and assumptions about what combination of factors are motivating Irish-trained health professionals to emigrate need to be explored and tested.

In Ireland, as elsewhere, the economic recession has resulted in salary reductions and tax increases [26], along with cost-containment measures across the health system. To what extent has the economic recession been a trigger for health professional emigration? How important have recession-related measures, such as reduced entry level salaries for health professionals, been in triggering emigration and making them reluctant to return home? To what degree is health professional emigration indicative of a wider set of problems in how a country manages its health workforce and broader health system? Oulton notes that employers in many countries have failed to address long-standing deficiencies relating to working hours, training, staffing levels and salaries [39]. Previous research by the authors has recommended a greater emphasis by employers and health workforce planners on retention than international health professional recruitment as a response to staffing shortages [15].

Drawing on qualitative and quantitative data from 388 Irish-trained emigrant health professionals, this paper seeks to improve understanding of the dynamics of health professional emigration from the perspective of the emigrants themselves. Qualitative data will provide insight into their motivations for emigration and their perspectives on whether or not they plan to return to work in the Irish health system. This information will be beneficial to health workforce planners in Ireland and other high-income source countries and should help to inform improved health workforce policy and practice.

## Methods

A mixed-method online survey of health professionals (doctors, nurses and midwives) who had recently emigrated from Ireland was conducted in July/August 2014, using convenience sampling. There were 556 responses to the survey, of which 388 were completed responses. The research was designed as a pilot to inform the development of a large-scale project on health professional emigration. Ethical approval for this study was obtained from the Royal College of Surgeons in Ireland in early 2014. This paper presents qualitative findings from that online survey.

### Survey design

The survey tool was designed drawing on the wider literature on health professional migration as well as the authors’ previous experience of conducting surveys with non-EU migrant doctors and nurses [15, 40] and with nurses and midwives [41]. The survey contained 21 questions, including 7 which allowed free-text responses. In addition to the questions analysed in this paper, free-text response questions were also used to gather data on the main reasons for emigration, the factors that attracted respondents to a specific destination country and the factors that might encourage them to remain there. Although most of these free-text responses were subsequently categorised and quantified, collecting free-text responses allowed for all factors influencing respondents’ emigration decisions to be identified. The survey was piloted with a number of health professionals prior to its launch online.

### Survey recruitment

Emigrant health professionals are a hard-to-reach group. For academics conducting research with emigrants, ‘*the lack of a representative sampling frame . . . has proved a major stumbling block*’ [6]. This project initially sought to access a representative sample of emigrant health professionals by sampling from the verification records held by the relevant source country professional councils. Unfortunately, it was not possible to access a representative sample in this way. It was decided instead to recruit a convenience sample of emigrant health professionals using health professional contacts and social networking sites as gatekeepers. This was an online form of snowball sampling whereby emails were forwarded and/or Facebook posts issued by the research team via gatekeepers, inviting emigrant health professionals to participate in the survey. While not a representative sample, it was felt that using snowball sampling to access a convenience sample would provide valuable insights into health professional emigration and would inform the subsequent development of a larger-scale study on health professional emigration. The recruitment process is described more fully in a separate manuscript (McAleese et al.: Gone for good? A survey of emigrant health professionals, submitted).

### Survey and data analysis

In line with the literature, free-text questions were incorporated in the survey so as to obtain information on all factors influencing emigration and also to provide respondents with an opportunity to voice their opinions [42]. Data generated from these questions were imported from SPSS into MaxQDA where the data were manually coded, using thematic coding [43]. The main themes to emerge from the question on health system change included contracts/salaries, better working conditions, health system reform and training and career progression. The main themes to emerge from the final ‘any other comment’ question included health worker emigration and health system reform. The analysis of free-text survey responses differs from analysis of qualitative interview data in having a small amount of data from a large number of respondents—less depth, but from a greater breadth of respondents. *Verbatim* quotes from respondents were used to illustrate themes [42]. The presentation of qualitative findings sought to follow the recommendations made by Sandelowski [44]. Respondents are referred to in text as RNXXX, meaning Respondent Nurse and their survey number; RDXXX, Respondent Doctor; and RMXXX, Respondent Midwife.

## Findings

### Respondent profile

Of the 388 emigrant health professional respondents eligible to participate in the survey, 307 were doctors, 73 were nurses and 8 were midwives. The majority (89%, *N* = 336) of respondents were Irish-trained, and most (93%, *N* = 338) had emigrated from Ireland since 2008. Most respondents (58%, *N* = 200) were 25-34 years old, with a further (31%, *N* = 108) aged 34–44 years. Females accounted for 58% (*N* = 203) of respondents. Most respondents had emigrated to Australia (33%, *N* = 115), the UK (29%, *N* = 103) and the USA (17%, *N* = 59).

Respondents were asked to identify the grade at which they last worked in the Irish health system to assess seniority prior to migration. Among medical respondents, 90% (*N* = 274) had worked as junior hospital doctors^j^ prior to emigration. Of these, 22% (*N* = 85) had worked at Specialist Registrar level (the most senior level of junior hospital doctor) and 20% (*N* = 77) at intern level (the most junior level of junior hospital doctor). Only 4% (*N* = 11) of respondents had worked at Consultant level and 6% (*N* = 19) at GP level prior to emigration. Among nursing and midwifery respondents, 74% (*N* = 56) had worked at Staff Nurse/Midwife level and 17% (*N* = 13) had been students prior to emigration.

### Questions analysed

This paper presents an analysis of responses to two open-ended questions. A high proportion of respondents answered these open-ended questions—343/372 (92%) responded to the question, ‘What changes to the Irish health system might attract back emigrant doctors, nurses or midwives?’, with responses ranging from 2 to 177 words, and 209/372 (56%) respondents answered the final survey question: ‘Do you have any other comments about the emigration of health professionals from Ireland?’, with responses ranging from 1 to 297 words.

The themes to emerge from the two questions overlapped and are presented together. The findings tell the story of a large group (*N* = 388) of health professionals who had emigrated from a high-income country—the Republic of Ireland. They discuss their reasons for emigration, with many seeing it as a means of escaping from difficult working conditions in Ireland, their source country. They describe a lack of respect afforded to health professionals in the Irish health system, particularly in relation to staffing levels and working conditions. Respondents spoke of the superior working conditions in their destination countries, which appeared to both vindicate their emigration decision and complicate the decision to return. The key take-home message from this paper is that any measures to improve retention or encourage reform are incomplete without considering the perspective of emigrant health professionals. Although findings presented in this paper suggest that system-level change is necessary to improve working conditions in the Irish health system, change must respond to and be informed by the experiences of individual health professionals.

### Working conditions driving emigration

Respondents felt that their emigration from Ireland had been driven by professional rather than personal reasons. Of the top five reasons for emigration given by respondents, all but one related to the workplace (in order of preference: working conditions, training, career progression, financial reasons, personal reasons). Given the impact of economic recession on Irish households generally in terms of unemployment, negative equity and debt burdens, this was a surprising finding.

Respondent health professionals, particularly doctors, felt that the working conditions experienced in Ireland left them with ‘*no option but to leave*’ (RD44). Both nurse and doctor respondents gave concrete examples of the working conditions they had experienced in the Irish health system, particularly in relation to long working hours and described the impact on their lives.

‘*I frequently worked 36+ hour shifts and almost always more than 80 hours/week*’ (RD211).

‘*In emergency you might have one day off here and there. There’s no real pattern from a life planning point*’ (RD283).

‘*No point having days off when you have to spend the whole time recuperating from the exhaustion of your working days*’ (RN48).

Respondents were acutely aware of the impact these working conditions had on the health and well-being of the health workforce generally and on themselves specifically. One respondent spoke of the impact of working conditions on their own well-being:

‘*I ended up in hospital twice, because of the ridiculous amount of work we did due to long hours and under staffing*’ (RD127).

Respondents described their emigration as a rational response to the working conditions experienced in the Irish health system.

### Disrespect and the Irish health system

Respondents felt that that health employers’ did not respect the health professionals in their employ and that poor working conditions were evidence of that disrespect. Respondents felt that this issue of respect, as reflected in improved health professional working conditions, needed consideration:

‘*it’s not about the money it’s about respect and understanding that we love working in medicine, but we love our families and health more*’ (RD283).

‘*It’s awful to feel exiled from your country because of the expectations and work conditions of your job*’ (RD299).

Respondents, especially doctors, spoke of a general disrespect for health professionals in Ireland, from the media and also from health employers. There was much discussion of an ‘*anti-doctor media narrative*’ (RD159) and a feeling that health professionals were regularly vilified in the media (RD19, RD94). Respondents felt that the Health Services Executive (HSE—the main public sector employer of health professionals) fuelled these campaigns in order to weaken the negotiating power of health professionals (RD57, RD206). It was noted that this was having a negative effect on health professional retention and also on the public’s attitude towards the health workforce, as one respondent explained:

‘*the HSE needs to start working with frontline health care staff instead of using the media to target us*’ (RD287).

### Nursing/midwifery experience

The experiences of nurses/midwifery respondents largely echoed that of their medical colleagues, particularly in relation to working conditions, staffing levels and the need for respect, other elements of their experience are presented here.

Nursing and midwifery respondents noted their dissatisfaction at the recent introduction of a graduate nursing scheme which sought to recruit entry level nurses on reduced rates of pay. All respondents who mentioned the scheme called for its abolition. One nursing respondent described this scheme as ‘*immoral . . . totally demoralising for the entire profession*’ (RN370). In relation to career progression more generally, nurses and midwives echoed their medical colleagues in calling for ‘*better career paths, more structure, improved staffing levels, pay scales which reflect the role*’ (RN313).

Nursing and midwifery respondents articulated the difficulties they faced in terms of their practice environment in the Irish health system, particularly as a result of staff shortages, and expressed concerns for patient safety.

‘*in certain areas in Irish hospitals, it is very difficult to demonstrate safe practice because of the pressure being put on staff nurses (staff shortages, increased workloads, etc.*’ (RN237).

‘*improve working conditions to make it safer for patients*’ (RN246).

There was a feeling that perhaps task shifting might enable the Irish health system to make better use of the available resources within its workforce, as this midwifery respondent explains:

‘*start using resources properly—use midwives to their full potential, e.g. using MLUs*^*k*^*on - site in hospitals, more midwifery led clinics, to free up our medical colleagues for the women who really need their input and care*’ (RM326).

### Emigration decision vindicated in destination country

If respondents had doubts about the decision to emigrate, these did not emerge strongly from the findings, with respondents expressing few regrets and describing the decision to emigrate as ‘*the best decision we ever made*’ (RD323) and as a ‘*no brainer*’ (RD323, RD208). The emigration decision appeared vindicated by the working conditions in the destination country. Invariably, the Irish health system compared poorly in comparison to the destination country, particularly in relation to training and working conditions.

‘*I was in shock when I first arrived to the UK when I saw how well doctors in training are supported and treated*’ (RD80).

‘*It was not until I worked abroad that I realised the full extent of this abuse. The absolute disregard for our training, lifestyles, good will is a disgrace*’ (RD292).

Emigration enabled respondents to realise that the difficult working conditions they had endured in Ireland were not universal. Respondents described the joy of working in a well-funded health system (RD260), of being appreciated and supported in their work (RD89), and working in less stressful environments (RN321, RD12) with less burnout and better morale: ‘*my colleagues are terrific and unbroken*’ (RD260). Respondents spoke about receiving more support and encouragement in their current health system, particularly from senior members of staff.

‘*When I got to Australia I immediately loved it. I loved the way the senior medical staff were friendly and helpful and encouraging and present on the floor (ED).*^*l*^*I loved the way I worked with nursing staff that felt and work like part of a team . . . I felt appreciated and could see a clear career path*’ (RD87).

The contrast in experience between destination and source country was also clearly articulated by nursing and midwifery respondents who felt more appreciated and respected in their destination country, ‘*in Ireland you work to live, here you live to work due to wonderful opportunities, training and job satisfaction*’ (RN305). The overall feeling was that there ‘*is no comparison between working at home and abroad*’ (RD379). Respondents gave many specific examples of things that were done differently in the destination country health system, ranging from tax incentives (RD41, RD186) to improved rotas and cover for sick leave/annual leave (RD22) to availability of research budgets (RD151). However, the main difference noted between the Irish health system and their current health systems appeared to be the staffing levels, as this respondent describes:

‘*I was shocked to come to work in a comparable unit in the UK . . . They have fully FIVE TIMES as many registrars, SHOs, interns, and consultants as the Irish hospital. It’s shocking when you look back*’ (RD127).

Improved working conditions in the destination country appeared to provide respondents with the opportunity to rediscover the joy of practising their profession without having to contend with a difficult work environment. This seemed both to vindicate the emigration decision and also complicate the decision to return.

### Return (personal vs professional motivations)

Despite their reservations about the Irish health system and their sense of anger at an emigration often perceived as involuntary, some respondents remained open to the possibility of return. In discussing their potential return to Ireland, respondents distinguished between personal and professional motivations. Many were eager to return ‘home’ to be near to family and friends:

‘*I would love to come back to Ireland due to having family and friends and the fact that it is my home*’ (RD59).

‘*I want to return home from a personal point of view but right now it makes no sense professionally*’ (RD74).

Others were eager to come home to help reform the Irish health system, but feared the professional costs associated with such a decision, as this respondent explains:

‘*I want to contribute to the recovery of the Irish healthcare system but I felt abused and demoralised as an intern and would need to see a significant improvement in the aforementioned areas before I would consider going back*’ (RD63).

Their experience in the destination country had shown respondents that a more pleasant working environment was achievable outside Ireland. These experiences were the lens through which any potential return to Ireland was seen. Respondents seemed to maintain a keen interest in developments in the Irish health system while abroad, which suggests an interest in returning to work in Ireland, which was contingent on evidence of improved working conditions there. Twenty-nine percent of respondents (*N* = 102) reported their intent to return to Ireland in the future with a further 29% (*N* = 103) open to the possibility.

### Reforms before return

Respondents felt that significantly improved working conditions in the Irish health system would be necessary prior to their return. The prospect of returning to the poor working conditions that they had left was simply not an option. The comparative experience of destination country working conditions, as well as a reflection on the working conditions that pre-empted their emigration, appeared to be key influencers in this regard:

‘*things must drastically improve for any to be attracted back to work in a significantly inferior health care system*’ (RD38).

‘*frankly I couldn’t bring myself to come back now to a system that is over stressed, understaffed and has ever worsening morale*’ (RD114).

The most common needed reforms cited by respondents (45%, *N* = 173) involved a combination of several factors, illustrating the complexity of health system reform and the challenges facing health workforce planners and policy-makers who seek to promote their return.

‘*Better working hours, transfer of tasks, support by senior colleagues, structured teaching, better pay, less hours, respect*’ (RD80).

‘*Improved working conditions and ability to provide good patient care—reduced working hours of junior doctors, more consultants, more ancillary staff, computerised health records, prompt access to tests and care for our patients*’ (RD344).

Staffing levels were considered an important dimension of health system reform, mentioned by 19% (*N* = 75) of respondents. Respondents highlighted the need for increased numbers of front-line staff, specifically in relation to improved nurse–patient ratios and ensuring adequate cover for staff on leave. They also spoke about the need for task shifting between health professionals to achieve a more appropriate use of existing staff resources.

‘*More nurses and doctors at the front - line, proper working hours without constantly having to stay late and do unpaid overtime due to the lack of adequate staff, resources available at hospital and ward level to allow staff to carry out their work without constant stress and worry. More appropriate staff to do jobs . . . reduction in the amount of documentation required from nurses that is not directly related to patient care*’ (RN225).

Although the reforms mentioned by respondents are wide-reaching, the underlying goal, as articulated by respondents, was for a safe practice environment in which health professionals could perform to the best of their abilities and ‘*to feel pride at the end of a shift well done instead of dismay at feeling that slap-dash substandard care has been provided*’ (RD115).

### The risk of non-return

While respondents discussed the possibility of return, they framed it as a time-limited window of opportunity. They felt that the Irish health system would have to move quickly to encourage the return of emigrant health professionals, before they become established (personally and/or professionally) abroad.

‘*Many of our friends are staying here until things improve at home. Worryingly the longer we are here the easier it is for us to stay*’ (RD368).

‘*if they don’t act quickly, the lost generation will settle and establish themselves elsewhere—and it’s harder to move back the longer you stay away*’ (RD217).

These insights demonstrate the heightened expectations of emigrant health professionals. Having experienced superior working conditions and practice environments in the destination country, they now expected similar conditions in the source country. Research findings also illustrate how ‘life’ can get in the way of a potential return, with respondents becoming more established (personally and professionally) in the destination country over time. Without specifically deciding to become permanent emigrants, respondents nevertheless spoke of the process through which the option of return becomes ever more remote.

‘*if terms and conditions at every level of medicine in Ireland are not changed soon, people will settle overseas, have children, etc. and won’t bother applying to return to Ireland regardless of terms. There is a relatively short window of opportunity*’ (RD151).

## Discussion

The overwhelming message from emigrant health professionals is that the unsatisfactory working conditions they experienced within the Irish health system were a major factor in their decision to emigrate and feature equally strongly in their considerations of whether or not to return. In this regard, the findings echo international findings [5, 45] and confirm previous findings in the Irish context [7]. In previous research by the authors, non-EU migrant doctors painted a ‘*bleak picture*’ [35] of working conditions in the Irish health system. Together, this growing body of research points to inherent (and interconnected) flaws in the Irish health system, flaws which probably contribute to high attrition of health professionals from the Irish health system and an over-reliance on internationally trained health professionals. The Irish health system has ‘*neglected more sustainable, long-term health workforce planning strategies*’ [15] for stemming the tide of health professional emigration and achieving health workforce sustainability.

As this paper has demonstrated, improved data collection is a prerequisite to a better understanding of health professional emigration from source countries such as Ireland and is a necessary first step in the process of addressing health professional emigration and moving towards health workforce sustainability, as recommended by the WHO Global Code [21]. In terms of administrative data, there is a need for accurate information on the active health workforce, those practising as distinct from those registered to practice. Data on the size and composition of the current health workforce (profession, place of employment, gender, age, grade, etc.) will enable a more accurate assessment of movements into and out of the health workforce. In terms of migration-specific data, there is a need for outflow data, i.e. details on all those who exit the Irish health workforce, as well as inflow data which also capture those who have re-entered the health workforce following time working in another country.

In the interim, more information could be requested from health professionals by the source country at the point at which they request verification of professional registration/good standing. If the source country were, at this point, to capture the verification request along with details of the individual health professional, their intended destination country and their intended duration of emigration, it would provide a valuable snapshot of health professional emigration intent *prior to* emigration.

Without more comprehensive, up-to-date routine data, source country health workforce planners are operating in a vacuum and face an uphill battle in seeking to address the emigration of health professionals. As the authors have stated previously, the lack of timely and comprehensive data is a serious impediment to workforce planning [16]. Poor health workforce data in relation to emigration make it difficult to (1) accurately ascertain the full extent of the emigration problem facing the health system, (2) demonstrate the drain on resources that health professional emigration represents, and (3) undertake medium- to long-term workforce planning, and finally, (4) the lack of baseline data will make it impossible to evaluate the effectiveness of any policy levers employed by the source country to address health professional emigration.

There have been some important policy responses to health professional emigration in recent years at the Irish and global levels. In 2007, Ireland increased the number of undergraduate medical places and introduced a new graduate entry medicine programme, resulting in an increase in the number of EU/Irish medical graduates produced (from 305 to 750 annually by 2015). According to policy predictions [46], Ireland is now training a sufficient number of doctors to meet demand. This is a significant achievement in a relatively short space of time and is recognised as an important component in the creation of a sustainable health workforce. According to the WHO Global Code, sustainability in terms of training must be accompanied by measures to retain sufficient health professionals to meet domestic demand [21]. A 2013/4 Strategic Review of Medical Training and Career Structure by Ireland’s Department of Health focused attention on the need to improve retention in the medical workforce [34, 47]. The 25 recommendations made in these reports address many of the broad medical recruitment and retention issues mentioned by respondents in this study. While these reports and recommendations are to be welcomed, the overwhelming message from respondent emigrant health professionals is that time is of the essence when it comes to implementation.

Ultimately, the solution to health professional emigration is retention, i.e. that source countries, such as Ireland, train and retain sufficient health professionals to staff their health system. Policies to encourage return, while important, are a reaction to, rather than a prevention of, health professional emigration. While some degree of health professional emigration is manageable, even desirable in terms of encouraging advanced specialisation and broadening horizons, it must take place in a managed fashion so that circular/return migration becomes the norm for emigrating health professionals, rather than the exception. In this paper, respondents spoke of emigrating for professional reasons—as a result of poor working conditions, a lack of respect, unclear career progression and poor practice environments. In the destination countries, respondents spoke of better working conditions, better morale and better staffing levels. If the Irish health system is to achieve a sustainable health workforce, then health professionals must be able to access good working conditions, training and career progression in the Irish health system. Emigration to achieve these basics must become a thing of the past.

## Conclusions

There are a few ‘silver linings’ for the Irish health system to take from this study of 388 emigrant health professionals. Firstly, emigrant health professionals remain interested in returning to their source country, which means that there is still a window of opportunity within which health system reform might provide the impetus to attract them back. However, this is a window that will narrow over time. Secondly, the reforms proposed by emigrant health professional respondents relate to endogenous factors within the control of the health system [48], more than to wider factors outside the control of the system, which in the Irish context might be economic recession, high unemployment and high levels of personal debt. A caveat, however, is that to attract back health professionals, the health system must reform in order to compete with the working conditions and career progression opportunities offered by other destination countries.

Finally, although the scale of the health system reform suggested by respondents is daunting, the prospect that health system reform might encourage the return of emigrant health professionals from the destination country, while also leading to improved retention in the source country, provides a strong impetus.

This paper highlights that doctors, nurses and midwives are emigrating from Ireland in search of better working conditions, clear career progression pathways and a better practice environment. The question for the source country is whether it can retain and attract back emigrant doctors, nurses and midwives by improving their working conditions.

### Limitations

A limitation of the study is that it uses a convenience sample which relied upon health professionals volunteering to participate in the survey. The use of social media supported by gatekeepers successfully recruited a large number of emigrant health professionals in a short space of time, with 40% (216) of responses received within 24 h of the survey going live (McAleese et al.: Gone for good? A survey of emigrant health professionals, submitted). The use of convenience sampling provided the research team with rapid access to ‘*a sufficiently large number of highly motivated respondents*’ [49]. One reason for the high response may have been a recent high-profile Facebook campaign, ‘Enough is Enough’, which sought improved working conditions for junior hospital doctors in Ireland. This meant that a large group of doctors, emigrants and non-emigrants, were already interested in and engaged on the survey topic, at the time the survey went live. There are two limitations of this approach. The first is that the sample over-represents doctors. The authors have attempted to counter that by presenting some findings specific to nursing/midwifery respondents, but accept that the paper affords more attention to the medical perspective. A second limitation is that accessing doctors via the ‘Enough is Enough’ campaign means that the sample may include a disproportionate number of doctors who were dissatisfied with the Irish health system.

Another limitation of the study is in its wider generalisability to other source and destination countries, that it focuses on Irish-trained health professionals, who, as English speakers, are perhaps more likely than their European counterparts to emigrate to (and integrate into) other English-speaking destination countries such as the UK, Australia, New Zealand, Canada and the USA. This makes the experiences of UK- and Irish-trained health professionals unusual in a European context although perhaps comparable to the emigration of francophone health professionals to Canada.

## Endnotes

^a^The Irish Medical Organisation (IMO) is the trade union representing doctors in Ireland.

^b^Consultants are the most senior grade of hospital doctor in the Irish context. They have completed their medical education and training, are registered on the Specialist register and have obtained the post of Consultant. NCHDs or junior hospital doctors are doctors who have graduated from medical school and are employed within the health service as Interns, Senior House Officers, Registrars, and Senior or Specialist Registrars. Some NCHDs are also involved in postgraduate training.

^c^Data presented in Table 1 comes from several sources, each of which collects and categorises data in different ways, making comparisons difficult. General practitioners (GPs) in Ireland operate as private businesses, but are contracted by the HSE to provide services to certain patient groups.

^d^In Figure 1, medical data are unavailable for 2008 and 2011.

^e^Some of those who opt to remain on the active nursing register may be involved in management, research and education. Others remain on the active nursing register although not working in direct nursing.

^f^The main trade union for nurses and midwives in Ireland.

^g^In February 2015, it was announced that 800 new nursing posts would be created in the Irish health system.

^h^A workforce database for junior doctors and consultants is currently under development by the National Doctors Training and Planning Unit, HSE and should be operational by 2016.

^i^Internship is the first clinical post after graduation from medical school. Internship (usually 1 year in duration) must be completed before full registration on the General Division of the Medical Register can be achieved.

^j^Known as NCHDs or non-consultant hospital doctors in the Irish context. NCHDs comprise all junior hospital doctors from interns, to Senior House Officers (SHOs), to Registrars, to Specialist Registrars (SpRs).

^k^Midwife-led units.

^l^Emergency department.

## Abbreviations

ED: Emergency Department
EU: European Union
EWTD: European Working Time Directive
HSE: Health Service Executive
IMO: Irish Medical Organisation
INMO: Irish Nursing and Midwifery Organisation
MaxQDA: Qualitative Data Analysis Software
MCI: Medical Council of Ireland
NCHD: Non-consultant hospital doctor (junior hospital doctor)
NMBI: Nursing and Midwifery Board of Ireland
OECD: Organisation for Economic Co-operation and Development
SPSS: Statistical Package for the Social Sciences
UK: United Kingdom
WHO: World Health Organization
